# Supplementary material for: A fungal pathogen manipulates phytocytokine signaling for plant infection
Source: Nat Commun. 2025 Nov 14;16:10021. doi: 10.1038/s41467-025-65934-2 (PMC12618502; doi:10.1038/s41467-025-65934-2)
Supplement: Supplementary file 1 — Supplementary Information [file 41467_2025_65934_MOESM1_ESM.pdf]

Supplementary Information

Title: A fungal pathogen manipulates phytocytokine signaling for plant infection

Authors: Chenlei Hua, Lisha Zhang, Annick Stintzi, Andreas Schaller, Hui-Shan Guo, Thorsten Nürnberger

Supplementary Figures

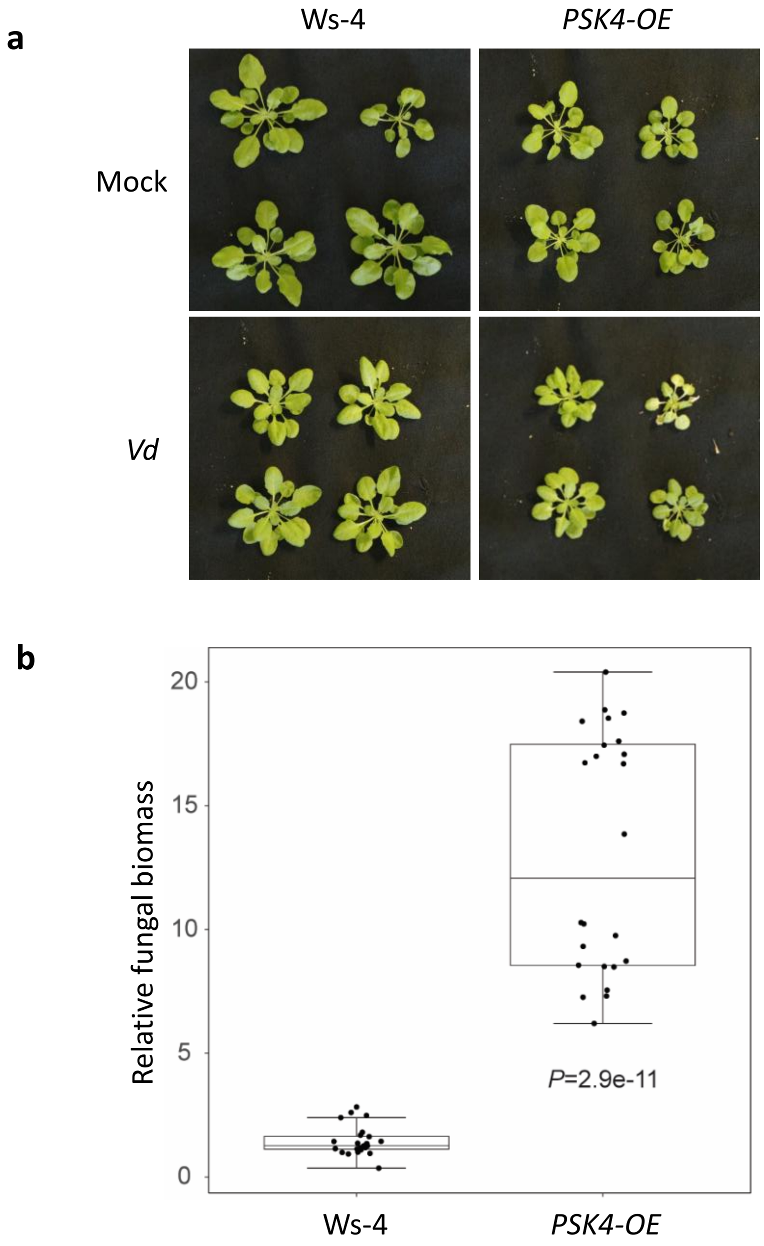

Supplementary Figure 1. **Arabidopsis wild type Ws-4 and PSK gene overexpressing line *PSK4-OE* infected by *Vd*. a**, Representative images of Arabidopsis Ws-4 and *PSK4-OE* infected with *Vd* at 20 days post inoculation. Water treatment served as mock controls. **b**, Quantification of *Vd* biomass in infected Arabidopsis plants from panel A. DNA was extracted from whole plants above the soil, and the relative fungal biomass was assessed by comparing Ct values of *Vd* actin and Arabidopsis *EF1α* genes. The experiment was repeated 3 times (n=24). Statistical difference between Ws-4 and *PSK4-OE* was analysed using a two-tailed Student's *t*-test.

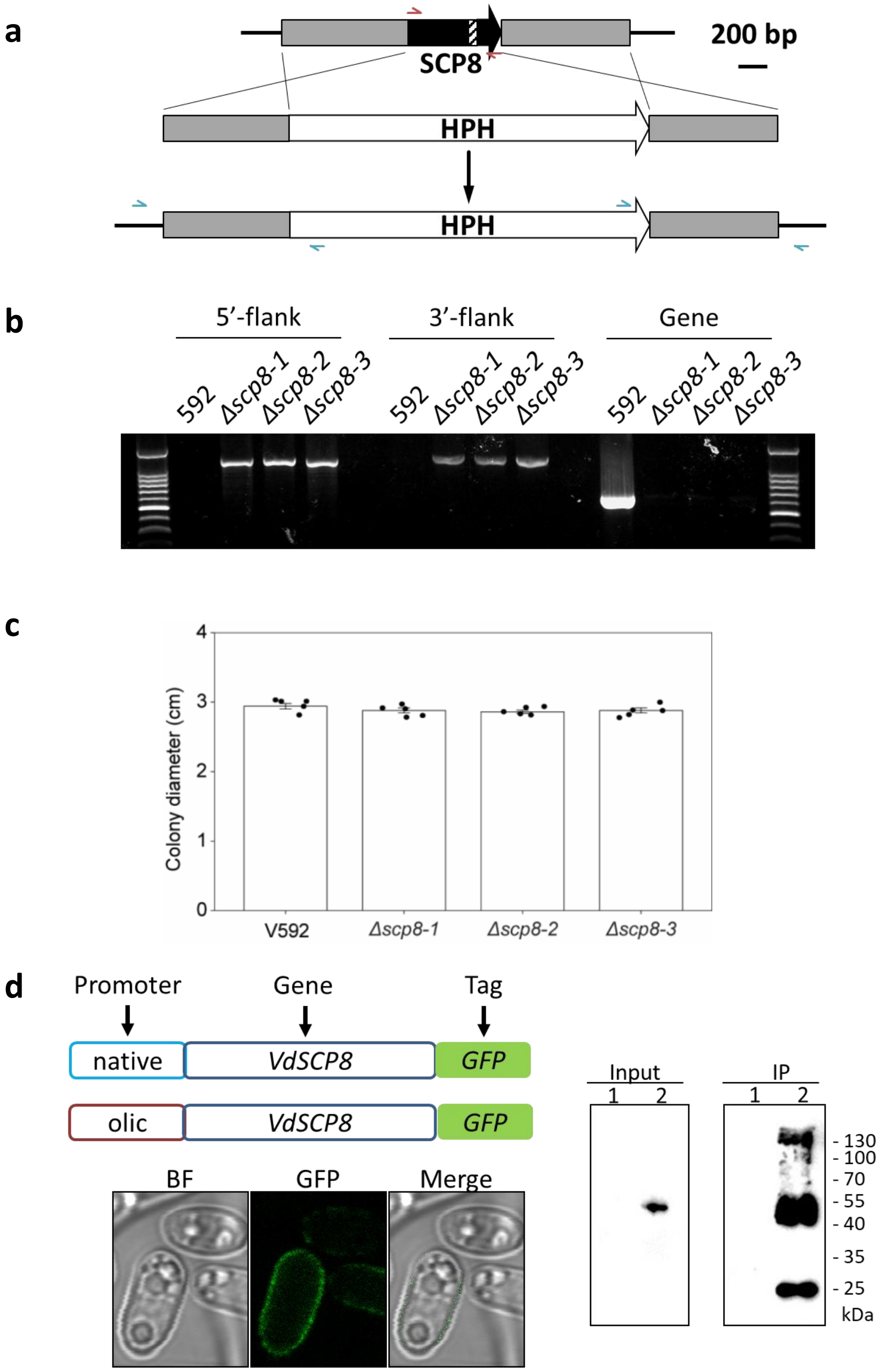

Supplementary Figure 2. **SCP8 gene knockout and complemented transformants. a**, Strategy for SCP8 gene knockout. **b**, Verification of SCP8 gene knockout mutants by genomic PCR. **c**, Colony diameter comparison of *Vd* wild-type strain (V592) and SCP8 knockout mutants *Δscp8-1*, *Δscp8-2*, *Δscp8-3* (n=5). **d**, Verification of SCP8 complemented transformants by GFP fluorescence observation and GFP antibody staining (1, *Vd* wild-type, 2, C-Polic strain). The experiment was repeated for 3 times with similar results.

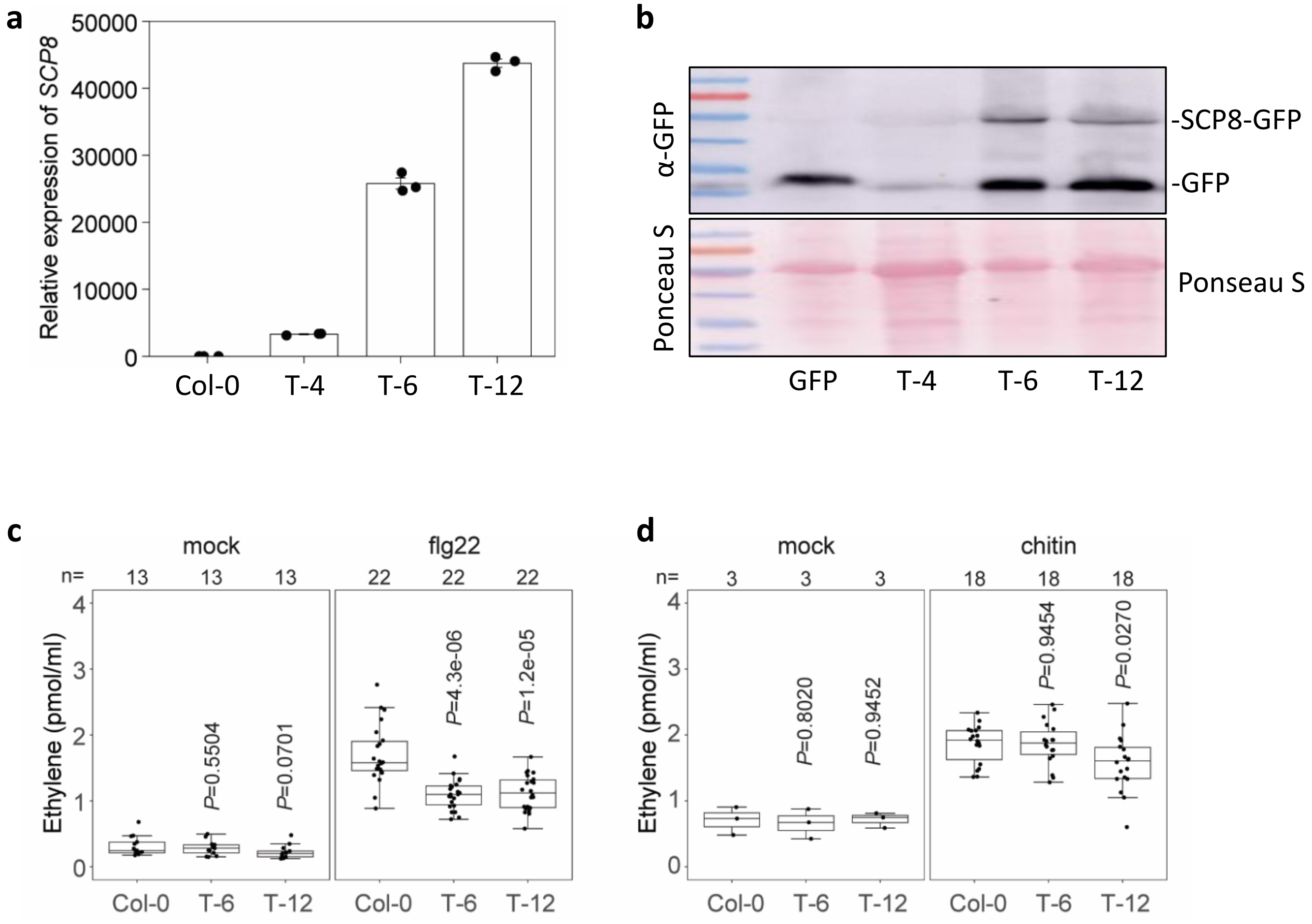

Supplementary Figure 3. **Transgenic Arabidopsis expressing SCP8-GFP. a**, qRT-PCR analysis of SCP8 gene expression in Arabidopsis (wild-type Col-0, and transgenic lines T-4, T-6 and T-12, n=3). **b**, Protein blot analysis of Col-0 expressing free GFP (GFP) and SCP8-GFP transgenic lines (T-4, T-6, T-12) using GFP antibody. The experiment was repeated for 3 times with similar results. **c**, Ethylene production measured in Col-0, T-6 and T-12 leaves at 4 hours post-infiltration with 1 μM flg22. **d**, Ethylene production measured in Col-0, T-6 and T-12 leaves at 4 hours post-infiltration with 1 μM chitin. For **c** and **d**, statistical differences between Col-0, T-6 and T-12 were analysed using a two-tailed Student's *t*-test.

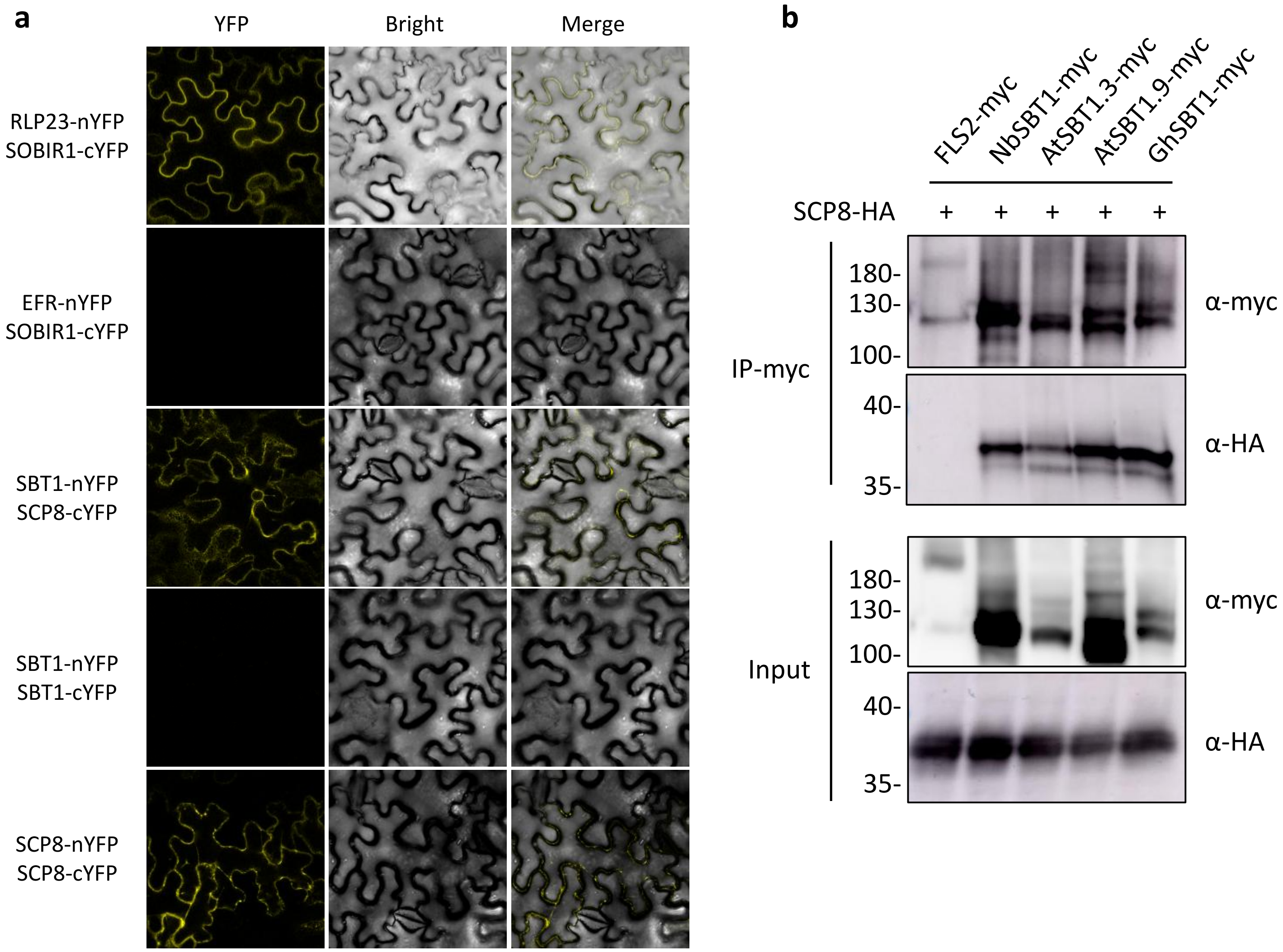

Supplementary Figure 4. **SCP8 interacts with plant subtilases as shown by BiFC and Co-IP assays. a**, Bimolecular fluorescence complementation (BiFC) analysis of the interaction between SCP8 and SBT1 in *Nicotiana benthamiana*. Agrobacterium strains carrying constructs expressing SBT1 fused to the N-terminus of YFP (SBT1-nYFP) and SCP8 fused to the C-terminus of YFP (SCP8-cYFP) were co-infiltrated into *N. benthamiana* leaves. As controls, SBT1-nYFP&SBT1-cYFP (a test for SBT1 homodimerization) and SCP8-nYFP&SCP8-cYFP (to test SCP8 self-interaction) were included. Fluorescence was observed at 48 hours post-infiltration using a Zeiss LSM880 confocal laser scanning microscope. YFP was excited with a 514-nm laser, and fluorescence emission was collected between 516–556 nm. Scale bar, 20 μm. Positive (RLP23-nYFP&SOBIR1-cYFP) and negative (EFR-nYFP&SOBIR1-cYFP) controls were included as described previously[7,21]. Images were processed with ZENblue software (Zeiss) for consistent adjustment of brightness and contrast. **b**, Co-immunoprecipitation (Co-IP) of HA-tagged SCP8 with myc-tagged plant subtilases. *N. benthamiana* leaves were co-infiltrated with Agrobacterium strains expressing SCP8-HA together with myc-tagged NbSBT1, AtSBT1.3, AtSBT1.9, or GhSBT1. Leaves were harvested two days after infiltration, and total proteins were extracted using RIPA buffer supplemented with protease inhibitors. Myc-tagged proteins were immunoprecipitated using Myc-Trap agarose beads (Chromotek) according to the manufacturer's protocol. Co-immunoprecipitated SCP8-HA was detected by western blotting using anti-HA antibody; expression of myc-tagged proteins was confirmed with anti-myc antibody. Co-infiltration with myc-tagged FLS2 served as a negative control. The experiment was repeated for 3 times with similar results.

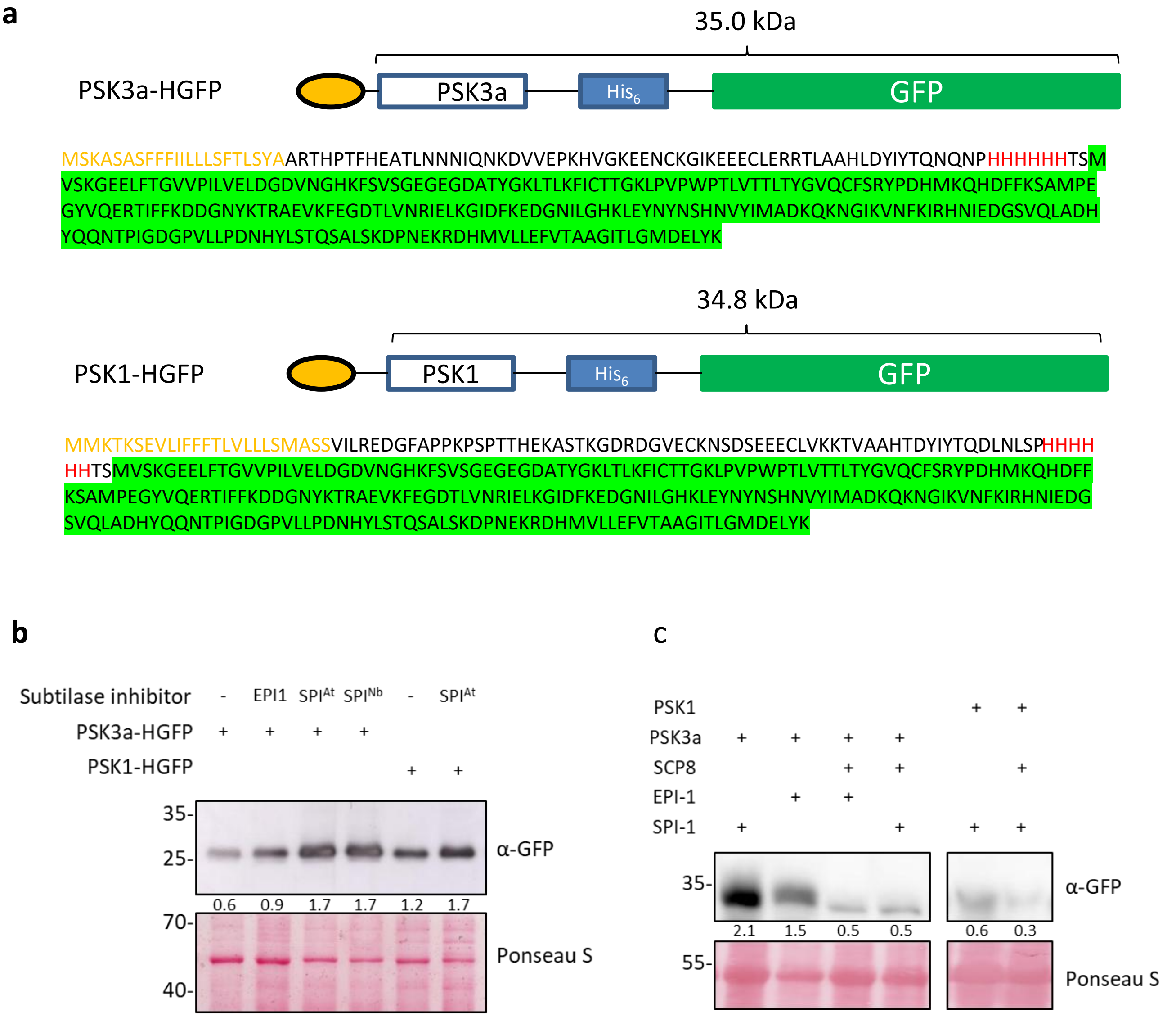

Supplementary Figure 5**. Transient expression of His- and GFP-tagged PSK precursors with subtilase inhibitors in *Nicotiana benthamiana*. a**. Structure and sequences of C-terminal His_6- and GFP-tagged *Nicotiana benthamiana* PSK3a (PSK3a-HGFP) and *Arabidopsis thaliana* PSK1 (PSK1-HGFP). **b**. Protein blot detection of PSK3a-HGFP and PSK1-HGFP in the presence of subtilase inhibitors including EPI1, SPI-1 from Arabidopsis thaliana (SPI^{At}), and SPI-1 homolog from *N. benthamiana* (SPI^{Nb}), in the apoplastic fluids of *Nicotiana benthamiana*. The relative protein levels were quantified by ImageJ software, normalized to corresponding loading control, and shown below the blot. The experiment was repeated for 3 times with similar results. **c**. Protein blot detection of PSK3a-HGFP (PSK3a) and PSK1-HGFP (PSK1) in the presence of subtilase inhibitors and SCP8 in *Nicotiana benthamiana*. Protein levels were quantified as described in (**b**). The experiment was repeated for 3 times with similar results.

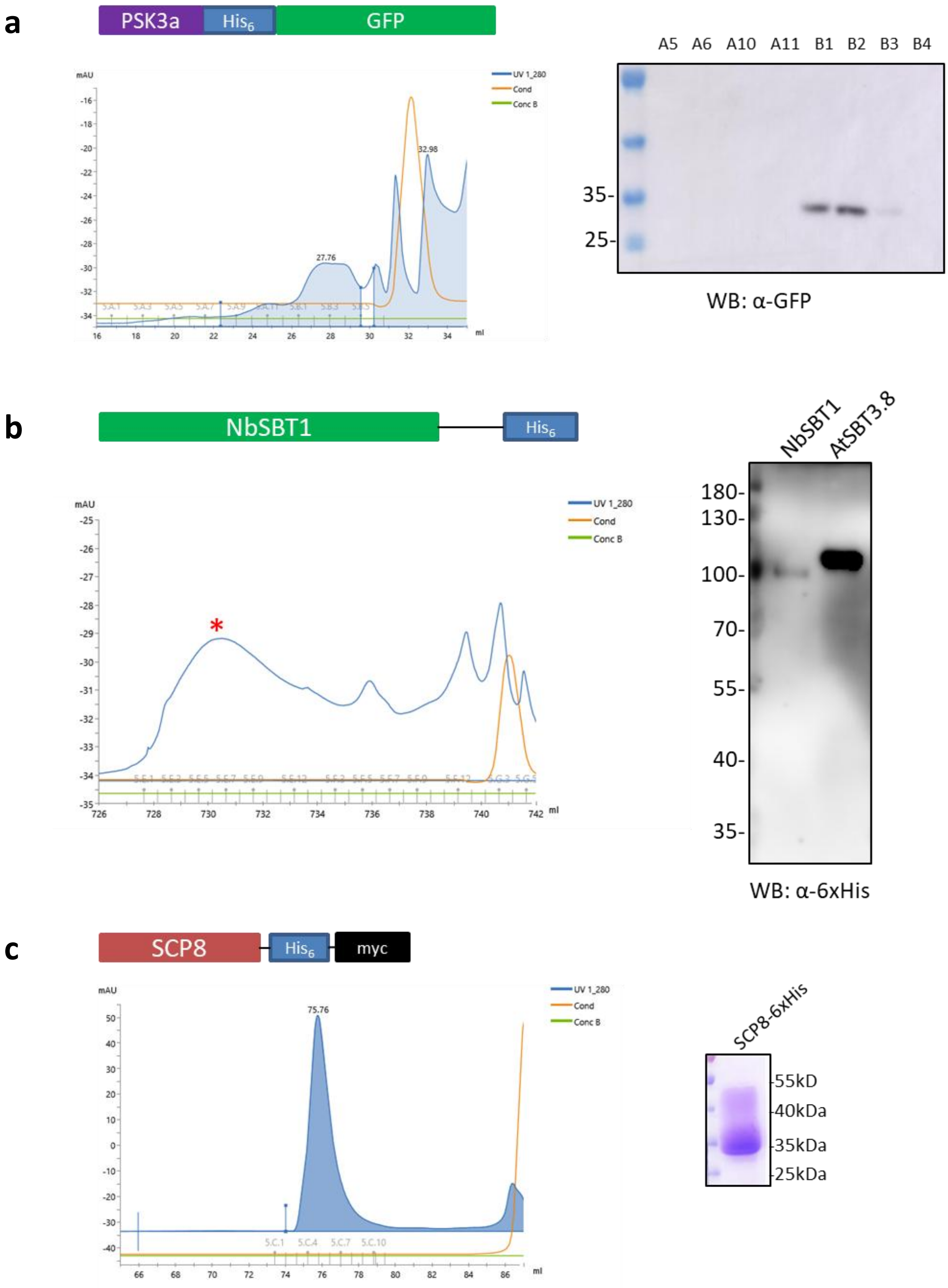

Supplementary Figure 6. **Protein Purification from *Pichia pastoris* and *Nicotiana benthamiana*.** **a**. Gel filtration of PSK3a-HGFP eluted from Ni-NTA beads, with fraction detection by protein blot using GFP antibody. **b**. Gel filtration of SBT1 eluted from Ni-NTA beads, with fraction detection by Protein blot using anti-His_6 antibody. **c**. Gel filtration of His_6-tagged SCP8 eluted from Ni-NTA beads, analyzed on SDS-PAGE and stained with Coomassie Brilliant Blue. All the experiments were repeated for at least 3 times with similar results.

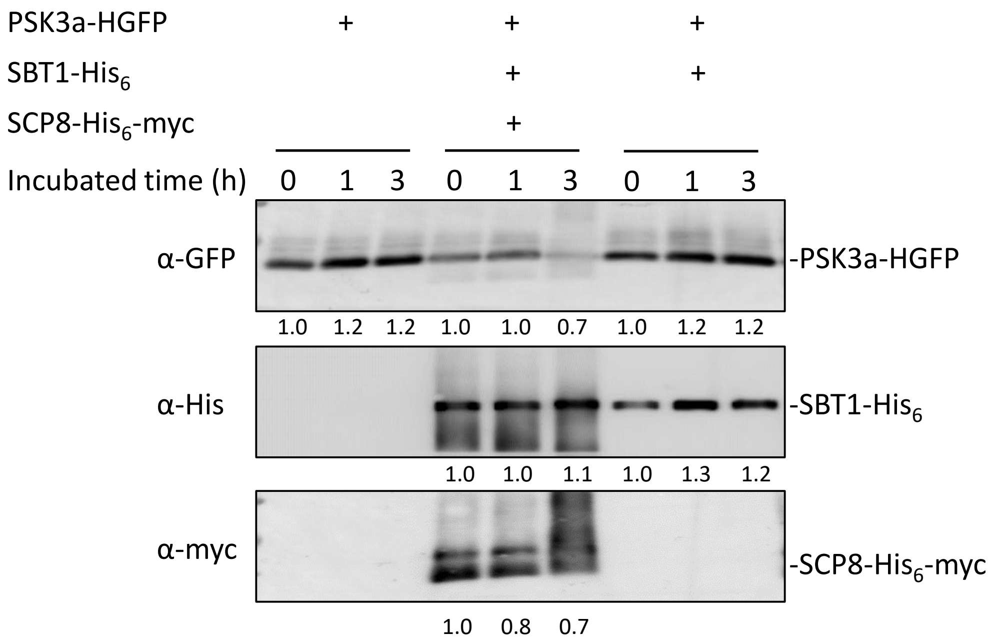

Supplementary Figure 7**. Digestion of PSK3a-HGFP purified from *Nicotiana benthamiana* (PSK3a).** Purified PSK3a-HGFP protein was incubated with either reaction buffer alone, a mixture of SCP8 (SCP8-His_6-myc) and SBT1 (NbSBT1-His_6), or SBT1 alone for up to 3 hours at room temperature. The incubated protein mixtures were detected by protein blot using anti-GFP, anti-His_6 and anti-myc antibodies. Approximate protein levels were determined by measuring band intensities from western blots using ImageJ software. For each condition, the band intensity at the 0 time point was set to 1.0 and the relative intensities were indicated below the blot. The experiment was repeated for 3 times with similar results.

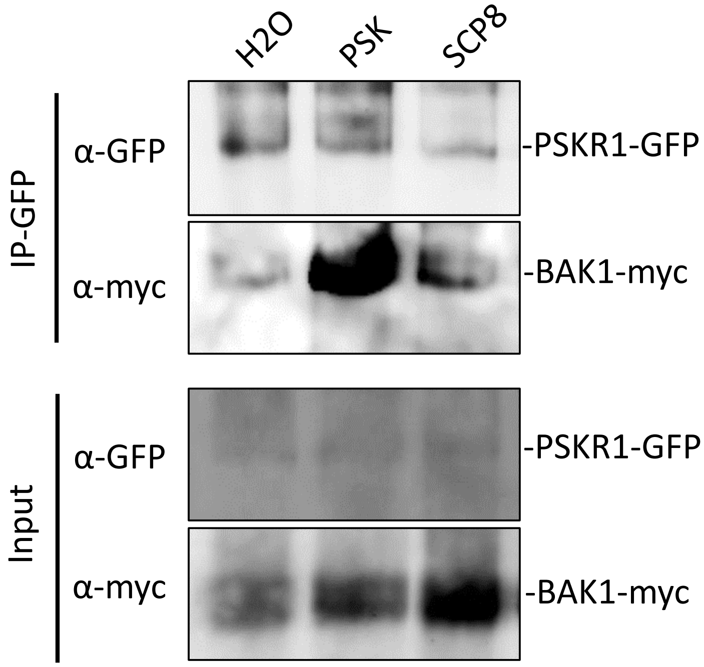

Supplementary Figure 8. **Protein blot of co-Immunoprecipitated protein samples from *Nicotiana benthamiana* co-expressing PSKR1-GFP and BAK1-myc.** Plants were infiltrated with H_2O, 1 µM PSK, or 2 µM SCP8, respectively. Input and GFP-Trap beads precipitated samples were detected by protein blot using anti-GFP and anti-myc antibodies. The experiment was repeated for 3 times with similar results.

Supplementary Table 1. **RNAseq data of *Nicotiana benthamiana* SOBIR1, PSKR1 and PSK-like genes.** Ten-day old seedlings of Nicotiana benthamiana was inoculated by two Verticillium dahliae strains, 171 (Rep-1) and 592 (Rep-2). Whole seedlings inoculated by V. dahliae were collected at 3, 6, 9, and 12 dpi for RNA isolation. Water treated seedlings were collected at 12 dpi for uninfected control (Mock). All the RNAs were sequences by BGI.

Gene Name	GeneID	Mock_RPKM	3 dpi_RPKM	6 dpi_RPKM	9 dpi_RPKM	12 dpi_RPKM	Replicate
PSKR1	NbS00021556g0001	5.950115622	12.77959734	16.03375249	22.52537377	19.73978334	Rep-1
			12.20242027	13.52219968	17.75924888	17.35568907	Rep-2
SOBIR1	NbS00033954g0001	2.621774155	12.98648362	77.44491859	72.36019598	77.06987526	Rep-1
			15.22970055	40.87074628	21.15632264	46.54998073	Rep-2
PSKR2-like	NbS00041770g0009	1.03394436	2.616700298	4.252327119	5.121764256	5.295797119	Rep-1
			2.88168887	4.720340947	3.71792031	5.426382127	Rep-2
SOBIR1	NbS00037616g0014	1.535634524	5.838647247	34.02336203	28.78351665	35.59121165	Rep-1
			6.150959074	18.95799942	8.192150166	25.82006644	Rep-2
PSKR2	NbS00035294g0005	1.449054353	2.791659108	2.616126189	3.279411396	3.533049461	Rep-1
			3.051494753	3.086523173	2.749541523	3.95292526	Rep-2
PSK-like	NbS00022281g0007	32.60498677	62.21451151	27.54806338	33.39726887	39.82364549	Rep-1
			51.63377085	32.05437264	29.60497543	39.04999159	Rep-2
PSK-like	NbS00047817g0002	23.19404709	2.497446981	9.406796504	15.07113669	5.412258982	Rep-1
			3.52000601	2.969281827	1.231924197	30.33434145	Rep-2
PSK-like	NbS00005245g0005	19.02684319	11.83380977	13.09635997	10.71616537	13.08522863	Rep-1
			12.74443319	19.63361861	14.8585143	10.4101204	Rep-2
PSK-like	NbS00031457g0009	18.95530038	46.26108263	40.88925595	57.49202278	44.12336882	Rep-1
			43.17467712	23.71381196	11.56104205	18.50054274	Rep-2

Supplementary Table 2. **Gene sequence information of this study**

NbPSK3a	ATGTCTAAAGCATCTGCCAGCTTTTTCTTCATCATCCTTCTCCTCTCTTTTACCCTTTCCTACGCTGCTCGCACTCACCCAACTTTTCAT<br>GAGGCCACCCTCAACAATAATATTCAAAACAAGGTATAACTCAAACTATTCAATAGCTTATTAACTTGTCAAAGGACGACACTCCGA<br>TCCTTCAATCATATATATTATGTTAATTCAATAGAAATGACATTATTGTTATGGATATTTGTTATACTCATTAATTAATAGGATTTTTTT<br>TTTTGAATTTATCTCAATTCTCAGGACGTTGTGGAACCAAAACATGTTGGTAAGGAAGAGAACTGCAAAGGAATCAAGGAAGAAG<br>AATGTTTGGAAAGGAGGACCTTGGCTGCTCATCTTGACTATATCTATACCCAAAATCAAAACCCCTGA
VdSCP8	ATGCACACCTCACTTCTCTCCCTCATGCTGGCCCCCTTGGCCCTCGCGAGCCCCATCCTCTCCCGCACCGATAAGCAGACCTGCTTCT<br>CAACAACCTCGGCTGTAAAGTCATGGGAGCTCAGATCCTTCGACTTCCACGCCAACTACATCTTCTCGACCCCATCGCACCAGAACT<br>CATGGGGTTACGTCTCCTTTGTCCTAGAAAACGCCAGTCTCGGCACCAAGACAAAATGCGAAGCCTCCAGCAGCAGCCTGAACGAC<br>TTTTTCTACGGCGATGTTGACTACCAGTGTGAGGCGCCAGAGGGAGACGTTCCCGAGGGTGCGCCTAGGTTGTCGCAGACCACGT<br>TCCGTTTCGACCGTCCCGGCGGAGAGGTCCAGGTCGCGCAGAGCTGGTATTGCTTCGACGACCCGCAGTTCCCGTCGAGGATCAC<br>GGCGAAGGGCGCAGAGCACGTCAAGCTCGACTGCGAGGAGACGTATACCGAGAATGGTAACTGGACCATTGGAGAGATCTACTC<br>GCAGAGGGACATCACCTGTGGCATTGTTGACCTGACTGTGCCGGTGAAGGAGATTGCAGCGGTAGCTTGA
FoSCP8	ATGCAATTCTTCACCCTTCTCTGCCTCGCCACCTCCGCCATGGCCCTCCCTCAGACTCTCACCAAGCGAGAGACTTGTATGGACAAG<br>GGCAGCAAGGTCACTGAATGGACTGTCAAGGACTTCAAGTACGAGGCCGTCTACACCCAAAACACTCCCACAAAGCAGACAAACT<br>CTGCCACCGTCACTTTCACCCTTCAGAACCGTGGTGTCGGTTATGAGGGCAAGTGCTCAGCCAAGTCCACAGACGCCAAGAAGGAT<br>TTCTTCACTGGTAACACCGACTACAACTGCGATGTTCCCTTCGAGGGCGACTCTGCTTCGTTCAAGTACAACCGCAAGTCCGGCGTC<br>ATCGCCATCTTCCAGCACTGGTCCTGCGTGAAGGAGGGCGGTTGGTACGAGGCCAAGGGCAACACAACCTTCACTCCCAAGTGTA<br>CCGAGAAGACCTGGAAGAACGCTCACTACAAGGCTGGCGGTGACAAGGCCTACTCCAACCGTCGGGTGACTTGCCAGCAGAAGC<br>AGCTCAAGGTTCCTGTCCTCGAGATGCAGGCTGTCCTGTAA
NcSCP8	ATGCTCACAACAACACTCACCACCGCCCTTGCGGCCCTCCTCCTCCCATTCTCCTCAGCTTCCCCCATCGCCGTCCGCCAGGATAATA<br>CAACTACCACAACTCCACCAACAAACCAAACCTCCTGCGGCGCCACCTCCTTTGGGAACTTTTCCTGGGCCCTCTCCGCCTTTGACT<br>ACCACGCCTCCTATATCTACACCAACCCCGCCCACCAAAACTCCTGGGGCTACGTCAACTTCAATTTGACCAACCCAGCCATCGGGC<br>CCTCGGTCACCACCGTCTGCAGCGCTTCAAGCAACCAGCTCAACGACTTCTTCTACGGGACCGTACAGTACACGTGCACCAACACG<br>CCCGCGGGCGTCAAGAGTGACGTGGGCAAGACCAAGTTTGATTTCAACCGGCCGACGGGGGAGTTGAGGTTCAACCAGAGCTGG<br>ACTTGCCGGGATGGGGATCCCAACTATCCTACCACGTTCACTGGATATGCGGCTGTCAAACTCCATTTGAACTGCACCGAGGCGAG<br>GTGGCAGAACCGGAGCTGGAAGCAGGGCCAGATTTACTCGACAAGGGATGTGAGGTGCTCGCCTGTTAATTTGAGTGTTGACCCC<br>AAGGAGATGACTGCTATGGCCTAG
NbSBT1	ATGCCTAATATCTTTGCTGATCACCATCATTGGCATTCTTCCACTATTGATTCCATTAAAGCTGCTGTTCCTTCATCAGTAGACAGATT<br>CCACTCTGCTCCAAAACTTGTTTACTCTTATGACAATGTATTTCATGGCTTCAGTGCTGTTTTGTCCAAAGATGAACTTGAAGCTTTG<br>AAGAAGTCACCAGGTTTTGTTTCAGCTTATAAAGATAGAACTGTGGAACCTCAAACTACCCACACATCTGATTTCCTTAAGCTTAAT<br>CCTTCATCTGGGCTATGGCCTGCTTCTGGTTTTGGCCAGGATGTGATCATTGGTGTTCTTGACTCTGGCATCTGGCCTGAATCTGTG<br>AGTTTTCGAGATGATGGTATGCCTGAAATCCCCAAAAGATGGAAGGGTATATGCAAGCCAGGCACACAGTTTAACACTTCATTGTG<br>CAACAGAAAACTTATTGGGGCTAATTACTTCAATAAGGGAATTTCGGCTAATGATCCAACTGTGAACATTTCCATGAATTCTGCAAG<br>GGATACTGATGGTCACGGCACACACGTTGCTTCCATTGCTGCTGGAAATTTCGCCAAAGGTGTTTCCCACTTTGGATATGCACCAG<br>GAACAGCAAGAGGAGTCGCGCCACGAGCTAGGCTGGCCGTATACAAGTTTAGCTTTAATGAAGGAACCTTTACTTCAGATTTAATT<br>GCTGCTATGGACCAAGCTGTGGCAGACGGTGTTGACATGATATCTATTTCATTTGGGTTCCGTTTCATTCCTTTGTATGAGGATTCT<br>ATATCCATTGCTTCTTTTGGAGCTATGATGAAGGGAGTGCTAGTTTCAGCTTCTGCTGGAAATCGAGGTCCGGGTATTGGAAGTTT<br>AAACAACGGATCTCCATGGATCTTGTGCGTGGCATCAGGCCACACTGACCGGACATTTGCTGGCACTTTGACTTTGGGAAATGGCT<br>TAAAAATCAGGGGTTGGAGCTTGTTTCCTGCAAGAGCCATTGTTAAGGATTCAACAGTGATTTACAACAAGACTCTAGCTGACTGC<br>AATTCGGAAGAATTATTATCACAACTATCTGATCCGGAACGTACCATCATTATATGTGAAGATAATGGGGATTTCTCTGATCAAATG<br>CGTATTGTCACTCGAGCAAGACTTAAAGCAGGCATCTTTATTTCTGAGGATCCAGGAGTGTTGGGATATGCTACATTTCCATGCCGT<br>GGAGTTGTGATTAACAAAAAGGAAGGAAACAAGTCTGCTACATTTCCCAACCCTGGAGTAGTGATTAACAAAAAGGAAGGAAAC<br>AAGTCATCAATTACGTAAAAAATACTGTCGATCCCACGGCCACCATCACATTCCAAGAGACGTATCTGGATGTAACACCAGCACCA<br>GTTGTTGCTGCATCCTCAGCACGAGGCCCCTCCAGAAGCTACTTGGGAATTGCAAAACCAGATATATTGGCACCAGGGGTGCTGAT<br>TCTTGCCGCATATCCACCAAACGTATTTGCTACAAGTATCGGGCCAAACATAGAATTGTCCACTGATTACATTCTTGAATCAGGCAC<br>ATCTATGGCTGCACCACATGCTGCTGGAATTGCAGCAATGCTAAAAGGCGCGCATCCTGAATGGAGTCCTTCAGCTATTCGCTCTG<br>CCATGATGACCACAGCAGATCCTTTGGACAACACTCGAAAACCTATTAAAGACTCGGACATTAACAAGGCTGCCACGCCCCTAGAC<br>ATGGGAGCAGGACACGTTGATCCCAACAGAGCGCTTGATCCTGGCCTAGTATACGATGCTACTCCACAAGATTACGTAAATCTTCT<br>ATGCTCTCTGAATTTCACAGAAGAGCAATTCAAGACAATTGCAAGATCATCAGACAACCACAACTGCTCAAATCCATCAGCCGATCT<br>CAATTACCCATCGTTCATTGCATTGTACCCCCTAGAGGGACCCTTCACATTGTTGGAACAGAAATTCAGGAGGACTGTTACAAATGT<br>TGGTAAAGGTGCAGCTACTTATAAAGCTAAGCTAAAAGCTCCAAAGAACACTACAGTTTCAGTATCACCACAGACTTTGGTATTCA<br>AGAAGAAAAATGAGAAACAAAGTTATACTTTGACAATTCGTTATTTAGGTGATGAGGGTCAAAGTAGAAACGTTGGGTCCATCACT<br>TGGGTTGAAGAGAATGGAAACCATTCTGTTAGGAGTCCTATAGTGACATCTCCCATTATTGAGATCTGGTCATAA

Supplementary Table 3. **Mass spectrometry analysis of SCP8 complexes in *Nicotiana benthamiana***

Category	Protein name	NCBI Access No.	Sol genomics ID	No. of detected peptides
Protease	**Subtilase (SBT1/3)**	ACA64703.1	Niben101Scf00726g02006.1	23
		ACA64705.1	Niben101Scf03154g03009.1	20
		ACA64704.1	Niben101Scf00726g02006.1	25
	Cysteine protease	AGV15820.1	Niben101Scf04007g01012.1	1
		ABW71226.1	Niben101Scf02763g05012.1	2
Pathogenesis-related	Chitinase	CAA55128.1	Niben101Scf06295g04023.1	6
		CAA35945.1	Niben101Scf07491g00003.1	2
	Osmotin	CAA46622.1	Niben101Scf09044g01012.1	3
		AAB24024.1	Niben101Scf09044g01012.1	2
		AAB32413.1	Niben101Scf09044g01012.1	2
	Peroxidase	AAK52084.1	Niben101Scf01453g06001.1	5
		P11965.1	Niben101Scf03460g04004.1	2
	Pathogenesis-related protein 4	CAA41438.1	Niben101Scf12045g06025.1	2
		CAA41437.1	Niben101Scf12045g06025.1	1
	NtPRp27	BAA81904.1	Niben101Scf03385g02011.1	5
	Elicitor inducible protein	BAB13708.1	Niben101Scf05326g00010.1	3
Signal transduction	Calmodulin	BAB61909.1	Niben101Scf03069g03001.1	5
		BAB61907.1	Niben101Scf02108g04004.1	3
	Extracellular Ca2+ sensing receptor	ABY57764.1	Niben101Scf18639g00026.1	3
	Calcium-dependent protein kinase	ADO79931.1	Niben101Scf03438g06004.1	4
	Calnexin-like protein	AIL30503.1	Niben101Scf03777g00018.1	8
	Calretulin	CAA59694.1	Niben101Scf00466g04036.1	6
	Cyclophilin	ABS30424.1	Niben101Scf01694g02008.1	5
		CAA78459.1	Niben101Ctg13380g00001.1	2
	phospholipase D	CAB06620.1	Niben101Scf12266g06004.1	7
		AAN04576.1	Niben101Scf02465g00004.1	3
	Leucine-rich repeat protein	ABD46739.1	Niben101Scf04386g04007.1	4
	Leucine-rich repeat extensin 1	BAF48665.1	Niben101Scf27793g02008.1	13

Supplementary Table 4. **Primers used in this study**

Number	Primer name	Sequence (5'-3')	Target	Purpose
For constructs in V.dahliae				
	VdSCP8 KO 5’ F	GGGTTTAAUCCTACTCTCTGCTGCTCGTTC	VdSCP8	VdSCP8 gene knockout
	VdSCP8 KO 5’ R	GGACTTAAUCGTGAGTGTGACGATGTGGAT	VdSCP8	VdSCP8 gene knockout
	VdSCP8 KO 3’ F	GGCATTAAUGGGGCTAAGCTATGCCAAAGC	VdSCP8	VdSCP8 gene knockout
	VdSCP8 KO 3’ R	GGTCTTAAUCCCTGTGATAGCTTCACAGTG	VdSCP8	VdSCP8 gene knockout
	Pnative_VdSCP8_F	ctatgaccatgattacgaattcCCAACATGGCTGGTGGTTGAG	Promoter of VdSCP8	VdSCP8 gene complementary
	VdSCP8_forgfp_R	gaaaagttcttctcctttactcatAGCTACCGCTGCAATCTCCT	VdSCP8	VdSCP8 gene complementary
	GFP5_forVdSCP8_F	AGGAGATTGCAGCGGTAGCTatgagtaaaggagaagaacttttc	GFP5	VdSCP8 gene complementary
	GFP5_R	cccgggtaccgagctcgaattcttatttgtatagttcatccatgcc	GFP5	VdSCP8 gene complementary
	VdSCP8_forPtrpC_F	CCTATTCTACCCAAGCATCCAAATGCACACCTCACTTCTCTCC	VdSCP8	VdSCP8 gene complementary
	VdSCP8_KO_5’-check_F	GCCTTGAGGAACAGTGATGAG	VdSCP8	VdSCP8 gene knockout
	VdSCP8_KO_3’-check_R	GAAATCAGCCTCCTTCCTCAAG	VdSCP8	VdSCP8 gene knockout
	Pnative-VdSCP8_pSul_F	cattattatggagaaactcgagCCAACATGGCTGGTGGTTGAG	VdSCP8	VdSCP8 gene complementary
	PAnoliC_pSul_F	cattattatggagaaactcgagtgtggagccgcattccgattc	from pLOB7	VdSCP8 gene complementary
	PAnoliC_R	GGAGAGAAGTGAGGTGTGCATgggcccttgtgatgtgatggagttgagat	from pLOB7	VdSCP8 gene complementary
	(PAnoliC)VdSCP8_F	atctcaactccatcacatcacaagggcccATGCACACCTCACTTCTCTCC	VdSCP8	VdSCP8 gene complementary
	VdSCP8(GFP)_R	tcctcgcccttgctcaccataagcttAGCTACCGCTGCAATCTCCT	VdSCP8	VdSCP8 gene complementary
	(VdSCP8)GFP_F	AGGAGATTGCAGCGGTAGCTaagcttatgagtaaaggagaagaacttttc	from pSul-GFP	VdSCP8 gene complementary
	GFP_pSul_R	tcagttaacggaattgggtaccttatttgtatagttcatccatgcc	from pSul-GFP	VdSCP8 gene complementary
	VdKO_5'check_R	AAATTTTGTGCTCACCGCCTGGAC	from pRH-HU2	VdSCP8 gene knockout
	VdKO_3'check_F	TCTCCTTGCATGCACCATTCCTTG	from pRH-HU2	VdSCP8 gene knockout
	SPpr1-VdSCP8_pSul_F	atctcaactccatcacatcacaagggcccatgggatttgttctcttttcaca	SPpr1-VdSCP8	VdSCP8 gene complementary
	SPpr1-VdSCP8_pSul_R	CTCCTCACCCTTGGAAACCATgggcccAGCTACCGCTGCAATCTCCTT	SPpr1-VdSCP8	VdSCP8 gene complementary
	HPH_cassette_F	cccatcgatgatcaggcctc	HPH	VdSCP8 gene knockout
	HPH_cassette_R	cagcactagtcgggggatc	HPH	VdSCP8 gene knockout
	PoliC_pSul_F	taaaacgacggccagtgccaagctttgtggagccgcattccgattc	oliC	VdSCP8 gene complementary
	PoliC_pSul_R	gatccagatctctgcagttaattaattgtgatgtgatggagttgagat	oliC	VdSCP8 gene complementary
	GFP_pSulC_F	actccatcacatcacaattaattaaggatccatggtgagcaagggcgagga	GFP	GFP gene expression
	GFP_pSulC_R	acgatctgcatccgaattcactagtttacttgtacagctcgtccatg	GFP	GFP gene expression
For constructs in planta				
	VdSCP8_pLOCGex_F	ccgtgcccaaaatCATCTCAGCCCCATCCTCTCC	VdSCP8	Protein expression
	VdSCP8_pLOCG_F	tcgacctgcaggcggccgcactagtATGAGCCCCATCCTCTCC	VdSCP8	Protein expression
	VdSCP8_pLOCGex_R	cctcgcccttgctcacCATCTCTCAAGCTACCGCTGCAATCT	VdSCP8	Protein expression
	Fo_pLOCGex_F	ccgtgcccaaaatCATCTCCCTCAGACTCTCACCA	FoSCP8	Protein expression
	Fo_pLOCGex_R	cctcgcccttgctcacCATCTCGAGCAGGACAGCCTGCAT	FoSCP8	Protein expression
	Nc_pLOCGex_F	ccgtgcccaaaatCATCTCTCCCCCATCGCCGTC	NcSCP8	Protein expression
	Nc_pLOCGex_R	cctcgcccttgctcacCATCTCGAGGGCCATAGCAGTCAT	NcSCP8	Protein expression
	NbSBT1_pDONR_F	GGGGACAAGTTTGTACAAAAAAGCAGGCTTCATGCCTAATATCTTTGCTGATCAC	NbS00041675g0001.1	Protein expression
	NbSBT1_pDONR_R	GGGGACCACTTTGTACAAGAAAGCTGGGTCTGACCAGATCTCAATAATGGGAG	NbS00041675g0001.1	Protein expression
	SCP8-BiFC_F	TCCGTCTCTcaccATGGGATTTGTTCTCTTTTC	VdSCP8	BiFC
	SCP8-BiFC_R	ATCGTCTCAccttAGCTACCGCTGCAATCT	VdSCP8	BiFC
	NbSBT1-BiFC_F	TCCGTCTCTcaccATGGGGTTGCCTTATTTTCA	NbS00041675g0001.1	BiFC
	NbSBT1-BiFC_R	ATCGTCTCAccttTGACCAGATCTCAATAATGG	NbS00041675g0001.1	BiFC

AtSBT1.3_pDONR_F	GGGGACAAGTTTGTACAAAAAAGCAGGCTTCATGGCTAACAAAAACCCACTTCAA	At5g51750	Protein expression
AtSBT1.3_pDONR_R	CCAACTTTGTACAAGAAAGCTGGGTCCAGAGGAGGCAACCATGTGAT	At5g51750	Protein expression
AtSBT1.9_pDONR_F	GGGGACAAGTTTGTACAAAAAAGCAGGCTTCATGGGGATGACCGTCGTAATTAT	At5g67090	Protein expression
AtSBT1.9_pDONR_R	GGGGACCACTTTGTACAAGAAAGCTGGGTCAGACTCTTGGACAAGGCTCGT	At5g67091	Protein expression
GrSBT_pDONR_F	GGGGACAAGTTTGTACAAAAAAGCAGGCTTCATGGGTCATCTGAACTATCTGC	Gorai.009G213500	Protein expression
GrSBT_pDONR_R	CCAACTTTGTACAAGAAAGCTGGGTCAGCCCCTAAGAAACCTAGCTC	Gorai.009G213500	Protein expression
NtSBT_pLOC_F	tcg acc tgc agg cgg ccg cac tag tat ggg gtt gcc tta tt	NbS00041675g0001.1	Protein purification
NtSBT_pLOC_R	cctcgcccttgctcaccatactagtTCAgtggtgatggtgatgatgTGACCAGATCTCAATAATGG	NbS00041675g0001.1	Protein purification
For constructs in Pichia pastoris			
SCP8_pPICZ_F	TTTGAATTCAGCCCCATCCTCTCCC	VdSCP8	Protein purification
SCP8_pPICZ_R	TTTGCGGCCGCAGCTACCGCTGCAATCTC	VdSCP8	Protein purification
GFP_pPICZ_F	TTTGAATTCatggtgagcaagggcgagg	GFP	Protein purification
GFP_pPICZ_R	TTTGCGGCCGCcttgtacagctcgtccatgccg	GFP	Protein purification
For RT-qPCR			
qVdSCP8_F	GGGCGCAGAGCACGTCAAG	VdSCP8	Gene expression analysis
qVdSCP8_R	ACCGGCACAGTCAGGTCAAC	VdSCP8	Gene expression analysis
qVdGAPDH_F	CGAGTCCACTGGTGTCTTCA	VdGAPDH	Gene expression analysis
qVdGAPDH_R	CCCTCAACGATGGTGAACTT	VdGAPDH	Gene expression analysis
qVdactin_F	GTCACCATGGTATCATGATTGG	VDAG_08445	Gene expression analysis
qVdactin_R	TTCTCCATGTCGTCCCAGTTG	VDAG_08445	Gene expression analysis
qAtEF1α_F	GGTGAAGATGACTCCAACCAAG	At1g07920/30/40	Gene expression analysis
qAtEF1α_R	CTCCGGTTGGGTCCTTCTTG	At1g07920/30/40	Gene expression analysis
qNbEF1α_F	GGTTAAGATGATGCCGACCAAG	NbS00019623g0001.1	Gene expression analysis
qNbEF1α_R	CGCCAGTTGGGTCCTTCTTG	NbS00019623g0001.1	Gene expression analysis
qNbSOBIR1_F	CCAGCAAGTCACAGAAGGGA	NbS00033954g0001.1	Gene expression analysis
qNbSOBIR1_R	GGCACTTCCAGATAATCCCATA	NbS00033954g0001.1	Gene expression analysis
qNbPSKR1_F	CTTTGGTGTCGTTCTTTTGGAG	NbS00021556g0001.1	Gene expression analysis
qNbPSKR1_R	CCAAGTAACTAATTGCTGAGAGG	NbS00021556g0001.1	Gene expression analysis
qNbPSK1_F	CAAAACAAGTTGGTAAGGACG	NbS00001285g0007.1	Gene expression analysis
qNbPSK1_R	TGATTTTGAGTATAGATATAG	NbS00001285g0007.1	Gene expression analysis
qNbPSK2_F	GGAATGTGATGATGGAGATGA	Niben101Scf05382	Gene expression analysis
qNbPSK2_R	GGGTGATTATGGTGTTGAGTG	Niben101Scf05382	Gene expression analysis
qNbPSK3A_F	CCAAAACATGTTGGTAAGG	NbS00047817g0002.1	Gene expression analysis
qNbPSK3A_R	GATATAGTCAAGATGAGCAGC	NbS00047817g0002.1	Gene expression analysis
qNbPSK3B_F	GAAAATTGAGATTAAGGAAGC	NbS00035636g0003.1	Gene expression analysis
qNbPSK3B_R	AAGATGAGCAGCCATTGTCC	NbS00035636g0003.1	Gene expression analysis
qNbPSK3C_F	AAAGTTAAAAATGATGAAGG	NbS00004726g0007.1	Gene expression analysis
qNbPSK3C_R	ATGAATGATCATTACTCTGAG	NbS00004726g0007.1	Gene expression analysis
qNbPSK4_F	AAGGGAAAATTGAGATTAAGG	NbS00035636g0003.1	Gene expression analysis
qNbPSK4_R	TAAATGTAATCAAGATGAGC	NbS00035636g0003.1	Gene expression analysis
qNbPSK5A_F	GTGTATATTTTTCAACAGCTC	NbS00031457g0009.1	Gene expression analysis
qNbPSK5A_R	TGTTGAGTATAAATGTAATCC	NbS00031457g0009.1	Gene expression analysis

qNbPSK5B_F	ATATTTTTCAACAGCTCATGG	NbS00022281g0007.1	Gene expression analysis
qNbPSK5B_R	TGTTGAGTATAAATGTAATCC	NbS00022281g0007.1	Gene expression analysis
