# Supplementary material for: A fungal pathogen manipulates phytocytokine signaling for plant infection
Source: Nat Commun. 2025 Nov 14;16:10021. doi: 10.1038/s41467-025-65934-2 (PMC12618502; doi:10.1038/s41467-025-65934-2)
Supplement: Supplementary file 2 — Reporting Summary [file 41467_2025_65934_MOESM2_ESM.pdf]

nature portfolio

Corresponding author(s): Thorsten Nürnberger

Last updated by author(s): Oct 24, 2025

Reporting Summary

Nature Portfolio wishes to improve the reproducibility of the work that we publish. This form provides structure for consistency and transparency in reporting. For further information on Nature Portfolio policies, see our Editorial Policies and the Editorial Policy Checklist.

Statistics

For all statistical analyses, confirm that the following items are present in the figure legend, table legend, main text, or Methods section.

n/a	Confirmed	
☐	☒	The exact sample size (*n*) for each experimental group/condition, given as a discrete number and unit of measurement
☐	☒	A statement on whether measurements were taken from distinct samples or whether the same sample was measured repeatedly
☐	☒	The statistical test(s) used AND whether they are one- or two-sided<br>*Only common tests should be described solely by name; describe more complex techniques in the Methods section.*
☒	☐	A description of all covariates tested
☒	☐	A description of any assumptions or corrections, such as tests of normality and adjustment for multiple comparisons
☐	☒	A full description of the statistical parameters including central tendency (e.g. means) or other basic estimates (e.g. regression coefficient) AND variation (e.g. standard deviation) or associated estimates of uncertainty (e.g. confidence intervals)
☐	☒	For null hypothesis testing, the test statistic (e.g. *F*, *t*, *r*) with confidence intervals, effect sizes, degrees of freedom and *P* value noted<br>*Give P values as exact values whenever suitable.*
☒	☐	For Bayesian analysis, information on the choice of priors and Markov chain Monte Carlo settings
☒	☐	For hierarchical and complex designs, identification of the appropriate level for tests and full reporting of outcomes
☒	☐	Estimates of effect sizes (e.g. Cohen's *d*, Pearson's *r*), indicating how they were calculated

Our web collection on statistics for biologists contains articles on many of the points above.

Software and code

Policy information about availability of computer code

Data collection	ZENblue for microscopy
Data analysis	Standard Excel software for RNA seq and mass spectrometry data, statistical data analysis; Adobe Illustrator for photo editing.

For manuscripts utilizing custom algorithms or software that are central to the research but not yet described in published literature, software must be made available to editors and reviewers. We strongly encourage code deposition in a community repository (e.g. GitHub). See the Nature Portfolio guidelines for submitting code & software for further information.

Data

Policy information about availability of data

All manuscripts must include a data availability statement. This statement should provide the following information, where applicable:

- Accession codes, unique identifiers, or web links for publicly available datasets
- A description of any restrictions on data availability
- For clinical datasets or third party data, please ensure that the statement adheres to our policy

The RNAseq data generated in this study have been deposited in the NCBI database under accession code GSE236729 [https://www.ncbi.nlm.nih.gov/bioproject/?term=GSE236729]. The protein mass spectrometry data generated in this study are provided in the Source Data file. All data generated or analysed during this study are included in this paper. Source Data are provided in this paper.

Research involving human participants, their data, or biological material

Policy information about studies with human participants or human data. See also policy information about sex, gender (identity/presentation), and sexual orientation and race, ethnicity and racism.

Reporting on sex and gender	*Use the terms sex (biological attribute) and gender (shaped by social and cultural circumstances) carefully in order to avoid confusing both terms. Indicate if findings apply to only one sex or gender; describe whether sex and gender were considered in study design; whether sex and/or gender was determined based on self-reporting or assigned and methods used. Provide in the source data disaggregated sex and gender data, where this information has been collected, and if consent has been obtained for sharing of individual-level data; provide overall numbers in this Reporting Summary. Please state if this information has not been collected. Report sex- and gender-based analyses where performed, justify reasons for lack of sex- and gender-based analysis.*
Reporting on race, ethnicity, or other socially relevant groupings	*Please specify the socially constructed or socially relevant categorization variable(s) used in your manuscript and explain why they were used. Please note that such variables should not be used as proxies for other socially constructed/relevant variables (for example, race or ethnicity should not be used as a proxy for socioeconomic status). Provide clear definitions of the relevant terms used, how they were provided (by the participants/respondents, the researchers, or third parties), and the method(s) used to classify people into the different categories (e.g. self-report, census or administrative data, social media data, etc.) Please provide details about how you controlled for confounding variables in your analyses.*
Population characteristics	*Describe the covariate-relevant population characteristics of the human research participants (e.g. age, genotypic information, past and current diagnosis and treatment categories). If you filled out the behavioural & social sciences study design questions and have nothing to add here, write "See above."*
Recruitment	*Describe how participants were recruited. Outline any potential self-selection bias or other biases that may be present and how these are likely to impact results.*
Ethics oversight	*Identify the organization(s) that approved the study protocol.*

Note that full information on the approval of the study protocol must also be provided in the manuscript.

Field-specific reporting

Please select the one below that is the best fit for your research. If you are not sure, read the appropriate sections before making your selection.

[x] Life sciences   [ ] Behavioural & social sciences   [ ] Ecological, evolutionary & environmental sciences

For a reference copy of the document with all sections, see nature.com/documents/nr-reporting-summary-flat.pdf

Life sciences study design

All studies must disclose on these points even when the disclosure is negative.

Sample size	Sample size was determined based on experimental trials and with consideration of previous publications on similar experiments to allow for confident statistical analysis. No statistical methods were used to predetermine sample size.<br>Previous publications considered to determine sample size:<br>Infection assay (https://doi: 10.1038/s41467-023-40384-w; https://doi: 10.1371/journal.pone.0015319; https://doi: 10.1101/gr.152660.112)<br>Ethylene accumulation (https://doi: 10.1038/nplants.2015.140.)<br>ROS assay (https://doi: 10.1038/nplants.2015.140.)<br>Gene expression (https://doi: 10.1073/pnas.0705306104)
Data exclusions	No data were exluded from the analyses provided.
Replication	Reproducibility of data was tested by multiple repetitions of the experiments described. All experiments were conducted at least 2 times or more as indicated on different days using biological materials produced independently (biological replicates). At least 3 technical replicates were included in the individual biological replicate experiments. Statistical evaluation was applied to all data sets obtained and is mentioned in figure legends when applicable. All attempts at replication were successful.
Randomization	Allocation of test plants used in our study was random. There was no targeted selection of individual plants for specific treatments.
Blinding	Blinding was not used in our study as it does not include clinical trials. The nature of the experiments conducted in our study requires that the experimentator knows precisely what plants have received what treatment. In plant, biology blinded/double-blinded studies are uncommon.

Reporting for specific materials, systems and methods

We require information from authors about some types of materials, experimental systems and methods used in many studies. Here, indicate whether each material, system or method listed is relevant to your study. If you are not sure if a list item applies to your research, read the appropriate section before selecting a response.

Materials & experimental systems

n/a	Involved in the study	
☐	☒	Antibodies
☒	☐	Eukaryotic cell lines
☒	☐	Palaeontology and archaeology
☒	☐	Animals and other organisms
☒	☐	Clinical data
☒	☐	Dual use research of concern
☐	☒	Plants

Methods

n/a	Involved in the study	
☒	☐	ChIP-seq
☒	☐	Flow cytometry
☒	☐	MRI-based neuroimaging

Antibodies

Antibodies used	anti-GFP, Torrey Pines Biolabs, Secaucus, New Jersey, US, Cat.-No. TP401, Lot 081211<br>anti-Myc, Sigma-Aldrich, St. Louis, Missouri, US, Cat.-No. C3956, Lot 094M4775V<br>Phospho-p44/42 MAPK (Erk1/2) (Thr202/Try204) antibody, Cell Signaling Technology, Beverly, MA, US, Cat.-No. 9101S, Lot 31<br>anti-BAK1, Agrisera AB, Vännäs, Sweden, Cat.-No. AS121858, Lot 1506-2<br>anti-His6, Cell Signaling Technology, Beverly, MA, US, Cat.-No. 12698S, Lot 4<br>anti-HA, Cell Signaling Technology, Beverly, MA, US, Cat.-No. 3724S,Lot 11
Validation	Relevant information on the antisera used in this study (including their applicability in plants/Arabidopsis) is found here:<br>anti-GFP: http://www.chemokine.com/Houston/rat&other/GFP.PDF<br>anti-Myc: https://www.sigmaaldrich.com/content/dam/sigma-aldrich/docs/Sigma/Datasheet/3/c3956dat.pdf<br>Phospho-p44/42 MAPK antibody: https://www.cellsignal.de/products/primary-antibodies/phospho-p44-42-mapk-erk1-2-thr202-tyr204-antibody/9101?site-search-type=Products&N=4294956287&Ntt=9101s&fromPage=plp&_requestid=1789178<br>anti-BAK1: https://www.agrisera.com/en/artiklar/bak1-bri1-associated-receptor-kinase.html<br>anti-His6:https://www.cellsignal.com/products/primary-antibodies/his-tag-d3i1o-xp-rabbit-mab/12698<br>anti-HA: https://www.cellsignal.com/products/primary-antibodies/ha-tag-c29f4-rabbit-mab/3724

Dual use research of concern

Policy information about dual use research of concern

Hazards

Could the accidental, deliberate or reckless misuse of agents or technologies generated in the work, or the application of information presented in the manuscript, pose a threat to:

No	Yes	
☒	☐	Public health
☒	☐	National security
☒	☐	Crops and/or livestock
☒	☐	Ecosystems
☒	☐	Any other significant area

Experiments of concern

Does the work involve any of these experiments of concern:

No	Yes	
☒	☐	Demonstrate how to render a vaccine ineffective
☒	☐	Confer resistance to therapeutically useful antibiotics or antiviral agents
☒	☐	Enhance the virulence of a pathogen or render a nonpathogen virulent
☒	☐	Increase transmissibility of a pathogen
☒	☐	Alter the host range of a pathogen
☒	☐	Enable evasion of diagnostic/detection modalities
☒	☐	Enable the weaponization of a biological agent or toxin
☒	☐	Any other potentially harmful combination of experiments and agents

Plants

Seed stocks	The Arabidopsis seeds pskr1-3, pskr1-3pskr2, and PSKR1-GFP transgenic line are from the labs of Dr. Birgit Kemmerling, Prof. Sebastian Wolf, and Prof. Klaus Harter, respectively, at Centre of Plant Molecular Biology (ZMBP), Eberhard-Karls-University of Tübingen, Tübingen, Germany. The detailed information of the seeds can be obtained from the publications below. pskr1-3: http://doi: 10.1111/tpj.12050; pskr1-3pskr2: http://doi: 10.1242/jcs.259134; PSKR1-GFP: http://doi: 10.1105/tpc.15.00306 The Arabidopsis bak1-5bkk1-1 seed is from the lab of Prof Cyril Zipfel at Molecular and Cellular Plant Physiology Department, University of Zürich. The detailed information of the seed can be obtained from the publication link: http://doi: 10.1371/journal.pgen.1002046
Novel plant genotypes	The Arabidopsis lines expressing SCP8-GFP were generated by transforming Col-0 with Agrobacterium tumefaciens GV3101 strain expressing pLOCGex::SCP8 by Floral Dip Transformation Method. The detailed method can be obtained from the link: http://doi: 10.1038/nprot.2006.97
Authentication	Expression of SCP8-GFP gene in Arabidopsis transgenic lines were detected by qRT-PCR and western blot with anti-GFP antibody.
